# Reduced-intensity post-cyclophosphamide-based graft-versus-host disease prophylaxis for umbilical cord blood transplantation: a single-center pilot study

**DOI:** 10.3389/fimmu.2026.1922972

**Published:** 2026-09-01

**Authors:** Yuntian Ding, Wei Xiao, Yaqun Wang, Haimei Deng, Hanchen Qin, Kexin Wang, Yin Wang, Dongjun Lin, Yunxin Zeng

**Affiliations:** Department of Hematology, The Seventh Affiliated Hospital, Sun Yat-sen University, Shenzhen, China

**Keywords:** ALLO = allogeneic, GvHD (graft-versus-host disease), hematology, PTCY, UCBT

## Abstract

**Background:**

Post-cyclophosphamide (PTCy)-based GVHD prophylaxis has shown significant success in haploidentical transplants. The potential of PTCy in adult umbilical blood transplantation (UCBT) remains unclear due to concerns about engraftment, given the limited number of CD34+ cells. This study aimed to explore the preliminary safety and efficacy of a reduced-intensity PTCy (RI-PTCy) in single-unit UCBT in adults.

**Methods:**

A total of 52 adult patients who underwent a single-unit UCBT were included in this pilot study. Patients were divided into three groups based on GVHD prophylaxis regimens: No PTCy (n=30), 100 mg RI-PTCy (n=15), and 400 mg RI-PTCy (n=7). Safety and efficacy were investigated post-transplantation.

**Results:**

The median times for neutrophil engraftment were 15 days (range, 12–27 days) in the No PTCy group, 16 days (range, 13–25 days) in the 100 mg RI-PTCy group, and 18 days (range, 15–24 days) in the 400 mg RI-PTCy group. Platelet engraftment occurred at 30 days (range, 21–77 days), 32 days (range, 18–40 days), and 31 days (range, 25–42 days), respectively. By day +100, the RI-PTCy groups had a significantly lower cumulative incidence of grade II-IV aGVHD than the No PTCy group. The cumulative incidence of grade II-IV aGVHD was 30.0% (95% CI, 13.6%-46.4%) in the No PTCy group, compared with 6.7% (95% CI, 0%-19.3%) in the RI-PTCy 100 mg group and 0% in the RI-PTCy 400 mg group (P = .044). Notably, the 400 mg RI-PTCy group presents a higher incidence of 28-day bloodstream infection. Moreover, the estimated 1-year GRFS were 53.3% (95% CI, 34.3%-69.1%), 71.1% (95% CI, 39.4%-88.3%), and 100.0% in the No PTCy group, the 100 mg and 400 mg RI-PTCy groups (P = .059).

**Conclusions:**

RI-PTCy appears promising as a GVHD prophylaxis strategy in this exploratory pilot cohort; these hypothesis-generating findings warrant validation in larger prospective studies.

## Introduction

Umbilical cord blood transplantation (UCBT) offers several advantages, including rapid availability, tolerance for multiple human leukocyte antigen (HLA) mismatches, absence of donor attrition, a potent graft-versus-leukemia effect, and a low incidence of chronic graft-versus-host disease (GVHD), making it an appealing option for patients with hematologic disorders (1, 2). However, Grade II-IV aGVHD occurs in approximately 20-50% of cases after single-unit UCBT, despite the administration of several GVHD prophylaxis regimens (3–6). Additionally, infections resulting from intensified immune suppression treatment due to aGVHD are collectively the leading causes of a higher rate of early non-relapse mortality (NRM) (7, 8). This emphasizes the urgent need for a more effective GVHD prophylaxis strategy and does not hinder engraftment, increase early toxicity, or compromise graft-versus-leukemia effect to improve outcomes.

Posttransplant cyclophosphamide (PTCy)-based GVHD prophylaxis has revolutionized allogeneic hematopoietic stem cell transplantation by reducing aGVHD while preserving favorable outcomes in engraftment, relapse, and survival (9, 10). However, its utility in the UCBT setting remains uncertain due to the limited number of CD34+ cells, which raises concerns about delayed engraftment or graft failure, especially at standard PTCy doses (50mg/kg on day +3 and day +4) in haploidentical hematopoietic stem cell transplantation (HSCT). A study evaluating the impact of PTCy in pediatric acute leukemia in the UCBT demonstrated the efficacy and safety of GVHD prophylaxis, but overall survival (OS) and relapse-free survival were lower than in the group that did not receive PTCy, especially at higher doses (11). Moreover, research has demonstrated that a single dose of PTCy (50mg/kg on day +3) effectively prevents aGVHD (12), suggesting that there might be potential to lower the PTCy dose further to align with the CD34+ cell count criteria in UCBT in adults.

PTCy has been linked to high incidences of aGVHD when used as a single agent (13). But regimens combining PTCy with calcineurin inhibitors (CNIs) or with CNIs and mycophenolate mofetil (MMF) have addressed these issues (9, 14). Furthermore, murine MHC-haplo HSCT studies indicated that an intermediate dose of PTCy offers better GVHD control than the traditional dose, with PTCy being most effective on day +4 (15, 16). Additionally, because of its superior engraftment and lack of mucosal damage, the combination of CNIs with MMF has become the preferred option for GVHD prophylaxis in the UCBT setting (6). Therefore, given this urgent clinical need, we designed a pilot study to evaluate the feasibility, safety, and preliminary efficacy of an RI-PTCy-based GVHD prophylaxis strategy compared with a CNIs and MMF prophylaxis strategy in adult patients undergoing single-unit UCBT.

## Patients and methods

### Patient eligibility

Between April 1, 2021, and May 10, 2026, fifty-two patients with hematopoietic disorders who underwent single-unit UCBT at the Seventh Affiliated Hospital of Sun Yat-sen University were included.

In a brief, 27 patients who received a single-unit UCBT between July 17, 2024, and August 4, 2025, were enrolled and randomly divided into three groups based on the GVHD prophylaxis regimen: 400 mg RI-PTCy (n=7), 100 mg RI-PTCy (n=15), and no PTCy (n=5), combined with the previous 25 adult patients who received UCBT without PTCy. The inclusion and exclusion criteria were reported in a previous study (17). Local institutional review boards approved this study. In accordance with the Declaration of Helsinki, all patients provided written informed consent.

### Transplantation procedures

All patients received a myeloablative conditioning regimen without total body irradiation, which consisted of fludarabine (Flu, 30 mg/m²/day for 4 days), busulfan (BU, 3.2 mg/kg/day for 4 days), cyclophosphamide (CY, 60 mg/kg/day for 2 days), with or without thiotepa (5 mg/kg for one day). The standard prophylaxis for GVHD includes cyclosporine and mycophenolate mofetil. RI-PTCy was administered intravenously at a dose of 400 mg or 100 mg on day +4. Granulocyte-colony-stimulating factor (G-CSF, 5-7 μg/kg daily) was initiated on day 5 and continued until neutrophil recovery. Rh-TPO or romiplostim was given subcutaneously at a dose of 300 U/kg (up to a maximum of 15,000 U) daily from day +5 to +19, and 250 µg once weekly from day +5 until platelet engraftment after UCBT, respectively. Specifically, Eltrombopag Olamine was administered to patients in the rhTPO group who presented with platelet counts below 10 × 10^9^/L on day +30 and who required additional platelet infusions. To prevent veno-occlusive disease (VOD), heparin was administered at a dose of 100 IU/kg daily until platelet counts dropped below 20× 10^9/L or when a high bleeding risk was present. Additionally, prostaglandin E1 was administered at 0.03 μg/kg per hour through continuous infusion, starting 12 to 24 hours before the conditioning regimen and lasting until day 28. Letermovir (LMV) was administered at a dose of 240 mg daily on day +14 to prevent cytomegalovirus (CMV) activation when available at our center. Additionally, intravenous immunoglobulin was given at a dose of 10 g weekly before neutrophil engraftment to prevent infection.

### Endpoints and definitions

The primary endpoints of interest were (1): the cumulative incidence of grade II-IV aGVHD within 100 days after transplantation, assessed based on the modified Glucksberg Criteria (18); (2) the cumulative incidence of moderate or severe chronic GVHD (cGVHD) within 1 year, according to the NIH Consensus Criteria (19); (3) one-year GVHD/relapse-free survival (GRFS). The secondary endpoints included the time to neutrophil and platelet engraftment, early immune reactions: pre-engraftment syndrome (PES), engraftment syndrome (ES), one-year OS, as well as the rates of early bloodstream infections, VOD, thrombotic microangiopathy (TMA), hemorrhagic cystitis (HC), and CMV, Epstein-Barr virus (EBV) within 100 days post-UCBT. Neutrophil and platelet engraftment were defined as the first day of three consecutive days with an absolute neutrophil count of ≥0.5 × 10^9/L and a platelet count of ≥20 × 10^9/L for seven straight days without transfusions. Primary graft failure was defined as the inability to reach an absolute neutrophil count of ≥0.5 × 10^9/L by day 28 after HSCT or as evidence of autologous reconstitution through chimerism analysis without any indication of disease relapse. Secondary graft failure was characterized by a decline in hematopoietic function and donor chimerism following initial engraftment. Bloodstream infections were identified through blood culture or metagenomics next-generation sequencing. OS was defined as the time to any cause of death post-transplantation. GRFS was defined as survival without grade III-IV aGVHD, moderate or severe cGVHD, or disease relapse or progression.

### Statistical analysis

Continuous variables were compared using the Kruskal-Wallis test, while categorical variables were analyzed with Fisher’s exact test. The cumulative incidence of neutrophil engraftment, platelet engraftment, 100-day aGvHD, and 1-year cGVHD was compared between groups using Gray’s test. Acute and chronic GvHD, as well as relapse, were analyzed with death as the competing event. Death in remission was treated as a competing event for relapse. The probabilities of OS and GRFS were estimated using the Kaplan-Meier method and compared across groups with the log-rank test. P values were two-sided, and results with P < 0.05 were considered significant. Analyses were performed with GraphPad Prism 10, version 10.4.0, and R version 4.3.1 (The R Foundation for Statistical Computing, Vienna, Austria).

## Results

### Patient and transplantation characteristics

The demographics and baseline clinical characteristics of the three groups are summarized in **Table 1**. The 100 mg RI-PTCy group is older than the No PTCy and 400 mg RI-PTCy groups (median ages: 52, 38, and 45 years), and male patients were more frequently included in the 100 mg RI-PTCy group. Over half of the patients in the No PTCy group had acute myeloid leukemia (AML), whereas only one patient with AML was involved in the 400 mg RI-PTCy group. All patients displayed a high disease risk index. Notably, five patients in the No PTCy group had CR2, no CR, or other statuses before transplantation. A relatively stronger myeloablative conditioning with thiotepa was observed in the 100 mg RI-PTCy group. Furthermore, over 50% of patients in the 100 mg RI-PTCy group had identical ABO compatibility. The three groups showed comparable levels of total nuclear cells. The number of CD34+ cells in the 100 mg RI-PTCy group was lower than in the other two groups. The prevalence of pre-existing anti-HLA antibodies among the groups: No PTCy group, 10.0% (3/30); 100 mg RI-PTCy group, 33.3% (5/15); and 400 mg RI-PTCy group, 14.3% (1/7). Notably, the donor-specific antibody in all patients was negative. All patients in the PTCy group received letermovir (LMV) prophylaxis, whereas 30% of patients in the No PTCy group did not receive LMV prophylaxis.

**Table 1:** Baseline demographics and clinical characteristics.

Characteristics	No PTCy n=30)	RI-PTCy 100mg (n=15)	RI-PTCy 400mg (n=7)
Age, y			
Median	38	52	45
Range	21-65	18-62	19-59
Gender			
Male	12 (40.0)	9 (60.0)	3 (42.9)
Female	18 (60.0)	6 (40.0)	4 (57.1)
Disease			
AML	17 (56.7)	7 (46.6)	1 (14.3)
MDS	4 (13.3)	4 (26.7)	3 (42.9)
ALL	9 (30.0)	4 (26.7)	1 (14.3)
Others	0	0	2 (28.6)
Disease status			
CR1	25 (83.3)	12 (80.0)	6 (85.7)
≥CR2	3 (10.0)	1 (6.7)	0
No CR or other	2 (6.7)	2 (13.3)	1 (14.3)
Disease risk			
High	30 (100)	15 (100)	7 (100)
Conditioning			
BFCY	13 (43.3)	1 (6.7)	4 (57.1)
TBFCY	16 (53.3)	14 (93.3)	3 (42.9)
Others	1 (3.3)	0	0
ABO compatibility			
Identical	10 (33.3)	8 (53.3)	1 (14.3)
Minor incompatibility	4 (13.3)	4 (26.7)	4 (57.1)
Major incompatibility	12 (40.0)	2 (13.3)	1 (14.3)
Bidirectional incompatibility	4 (13.3)	1 (6.7)	1 (14.3)
TNCs (× 10^7^ cells/kg)			
Median	2.56	2.64	2.49
Range	1.37-4.06	2.1-3.74	1.18-7.28
CD34 cells (× 10^5^ cells/kg)			
Median	1.43	1.29	1.44
Range	1.03-2.99	1.0-2.03	1.17-3.27
HLA matched			
6/6	1 (3.3)	2 (13.3)	1 (14.3)
5/6	8 (26.7)	7 (46.7)	2 (28.6)
4/6	21 (70.0)	6 (40.0)	4 (57.1)
HLA antibodies			
Presence	3 (10.0)	5 (33.3)	1 (14.3)
LMV prophylaxis			
Yes	21 (70.0)	15 (100)	7 (100)

AML, acute myeloid leukemia; ALL, acute lymphoblastic leukemia; MDS, myelodysplastic syndrome; CR, complete response; TNC, total nuclear cell; HLA, human leukocyte antigen; LMV, letermovir.

### Engraftment and chimerism

**Figure 1:**
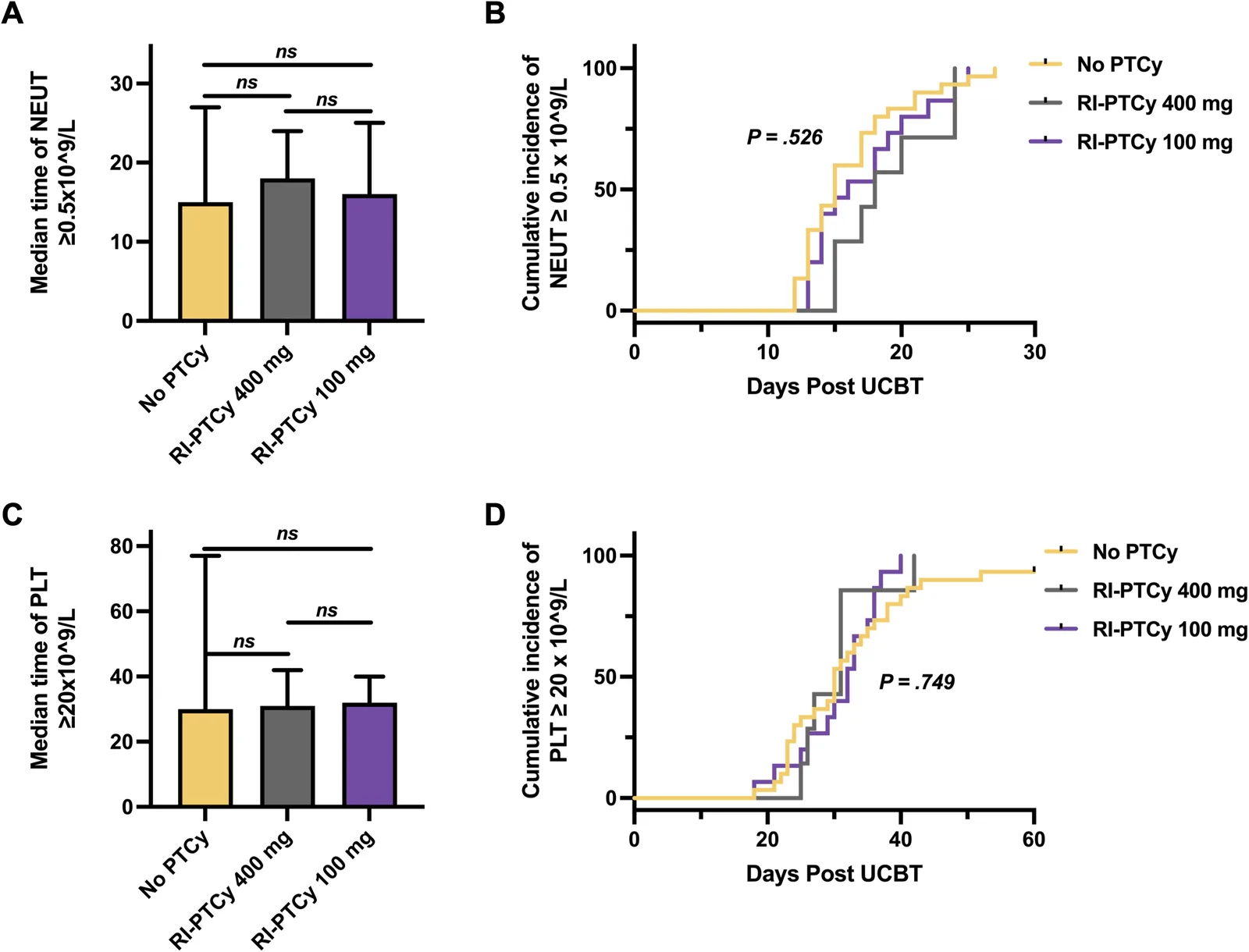
Engraft information in the No PTCy group (n=30), 100 mg RI-PTCy (n=15), and 400 mg RI-PTCy group (n=7). **(A)** Median time of neutrophil ≥0.5 x 10^9/L and **(B)** the cumulative incidence by day + 28 among three groups. **(C)** Median time of platelet ≥20 x 10^9/L and **(D)** the cumulative incidence by day + 60 among the three groups. ns, not significant.

All patients achieved neutrophil engraftment before day 28 after UCBT. The median neutrophil engraftment times were similar in all three groups: 15 days (range, 12–27 days) for the No PTCy group and 16 days (range, 13–25 days) for the 100mg RI-PTCy group. However, the neutrophil engraftment time in the 400mg RI-PTCy group was 18 days (range, 15–24 days), which is slightly longer than the other two groups but not significantly different (**Figure 1A**). The median PLT engraftment times were comparable across the three groups: 30 days (range, 21–77 days) for the No PTCy group, 32 days (range, 18–40 days) for the 100 mg RI-PTCy group, and 31 days (range, 25–42 days) for the 400 mg RI-PTCy group. The 60-day cumulative incidence of PLT engraftment was 93.3% (95% confidence interval [CI, 77.9%-99.2%) in the No PTCy group, while it was 100% (95% CI, 78.2%-100%) in the 100 mg RI-PTCy group and 100% (95% CI, 59.0%-100%) in the 400 mg RI-PTCy group. One patient achieved platelet engraftment on day +77, whereas another in the No PTCy group developed severe gastrointestinal (GI) aGvHD and TMA and failed to engraft. Furthermore, all patients reached full donor chimerism by day +30, with no cases of primary or secondary graft failure.

### Early immune reactions

**Figure 2:**
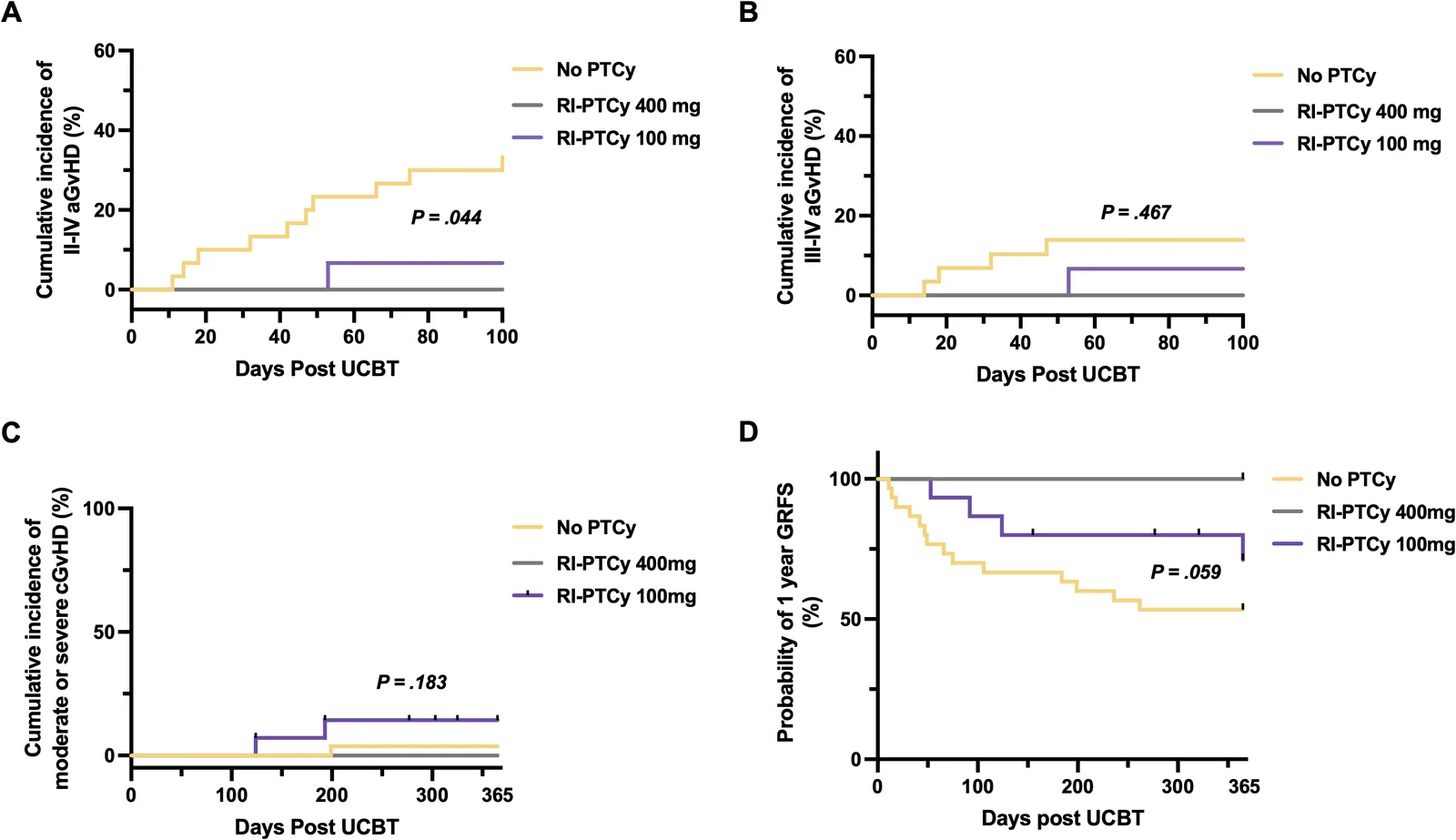
aGVHD, cGVHD cumulative incidences, and GRFS. **(A)** Cumulative incidences of grade II-IV and **(B)** grade III-IV acute GVHD by day +100. **(C)** 1 year of moderate or severe chronic GVHD. **(D)** 1 year of GRFS.

PES, ES, and aGvHD within 100 days were considered early immune reactions in UCBT. Seventeen patients (56.7%) in the No PTCy group developed pre-engraftment syndrome (PES), with a median of 7 days, compared to nine patients (60.0%) in the 100 mg group with a median of 8 days, and four patients (57.1%) in the 400 mg RI-PTCy group with a median of 7 days. Similar results were observed in the ES among the three groups: seven patients (23.3%), three patients (20.0%), and two patients (28.6%) in the No PTCy, 100 mg RI-PTCy, and 400 mg RI-PTCy groups (**Table 2**). A total of 15 patients (28.8%) developed aGVHD within 100 days, with a median onset of 47 days (range, 11–100 days). Grade II-IV aGVHD occurred in nine patients (30.0%) in the No PTCy group, one patient (6.7%) in the RI-PTCy 100mg group, and none in the RI-PTCy 400mg group. The skin (53.3%) and the gastrointestinal (GI) tract (40%) were the most frequently affected organs. Remarkably, all grade III-IV aGVHD cases were GI-aGVHD. At day +100, the cumulative incidence of grade II-IV aGVHD was 30.0% (95% CI, 13.6%-46.4%) in the No PTCy group, compared with 6.7% (95% CI, 0%-19.3%) in the RI-PTCy 100 mg group and 0% in the RI-PTCy 400 mg group (P = .044, **Figure 2A**). The incidence of grade 3 to 4 aGVHD was 13.3% (95% CI, 1.2%-25.5%), 6.7% (95% CI, 0%-19.3%), and 0% in the No PTCy, RI-PTCy 100 mg, and RI-PTCy 400 mg groups (P = .467, **Figure 2B**), respectively.

**Table 2:** Clinical and transplantation-related outcome.

End points	No PTCy (n=30)	100 mg RI-PTCy (n=15)	400mg RI-PTCy (n=7)
PES, no. of patients (%)	17 (56.7%)	9 (60.0%)	4 (57.1%)
ES, no. of patients (%)	7 (23.3%)	3 (20.0%)	2 (28.6%)
100-d csCMV reactivation, no. of patients (%)	5 (16.7%)	1 (6.7%)	0%
100-d csEBV, reactivation, no. of patients (%)	0%	0%	0%
28-d bloodstream infection, no. of patients (%)	10 (33.3%)	1 (6.7%)	4 (57.1%)
VOD, no. of patients (%)	0%	0%	0%
TMA, no. of patients (%)	3 (10.0%)	2 (13.3%)	1 (14.3%)
II-IV° HC, no. of patients (%)	5 (16.7%)	6 (40.0%)	2 (28.6%)

PES, pre-engraftment syndrome; ES, engraftment syndrome; CMV, cytomegalovirus; EBV, Epstein-Barr virus; VOD, veno-occlusive disease; TMA, thrombotic microangiopathy; HC, hemorrhagic cystitis.

### Infections, VOD, TMA, and HC

Five patients (16.7%) without LMV prophylaxis experienced csCMV reactivation within 100 days in the No PTCy group, while one patient (6.7%) experienced csCMV reactivation in the 100mg RI-PTCy group. None of the patients died of CMV-related organ damage. None of the patients developed csEBV reactivation within 100 days after UCBT. Ten patients (33.3%) in the No PTCy group, four patients (57.1%) in the 400 mg RI-PTCy group, and one patient (6.7%) in the 100 mg RI-PTCy group had bloodstream infections within 28 days after UCBT. Three patients (10.0%), one patient (14.3%), and two patients (13.3%) developed TMA in the No PTCy, 400 mg, and 100 mg RI-PTCy groups, respectively. None of the patients had VOD. The incidence of II-IV° hemorrhagic cystitis (HC) was five patients (16.7%) in the No PTCy group, two patients (28.6%) in the 400 mg RI-PTCy group, and six patients (40.0%) in the 100 mg RI-PTCy group (**Table 2**).

### Long-term outcomes

**Figure 3:**
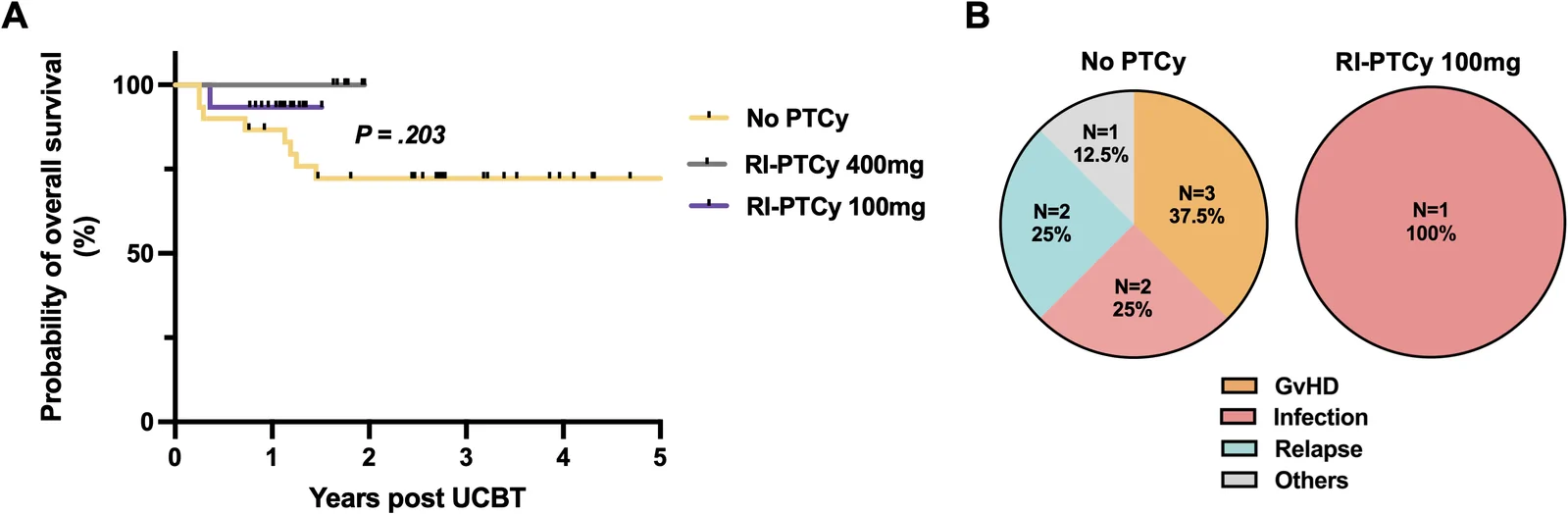
OS and cause of death in the No PTCy group (n=30), RI-PTCy group: 100 mg (n=15), and 400 mg (n=7). **(A)** Overall survival; **(B)** Cause of death.

For the No PTCy group, with a median follow-up of 38.2 months (range,9.2-61.3 months) for survivors, 23 patients (76.7%) were alive. In the 400 mg and 100 mg RI-PTCy groups, the median follow-up durations were 21.1 months (range, 19.6-23.4 months) and 13.4 months (range, 9.2-18.1 months), respectively. The estimated cumulative incidence of moderate or severe cGVHD at 1 year was 5.3% (95% CI, 0%-15.7%), 21.8% (95% CI, 0%-52.4%), and 0% in the No PTCy group, the RI-PTCy 100 mg group, and the RI-PTCy 400 mg group (P = .183, **Figure 2C**), respectively. For survival, the estimated 1-year OS was 86.5% (95% CI, 68.0%-94.7%), 93.3% (95% CI, 61.3%-99.0%), and 100.0% (P = .407, **Figure 3A**) in the No PTCy group, the 100 mg RI-PTCy group, and the 400 mg RI-PTCy group, respectively. The estimated 1-year GRFS were 53.3% (95% CI, 34.3%-69.1%), 71.1% (95% CI, 39.4%-88.3%), and 100.0% in the No PTCy group, the 100 mg RI-PTCy group, and the 400 mg RI-PTCy group, respectively (P = .059, **Figure 2D**). The differences were not statistically significant. Importantly, four patients (13.3%) in the No PTCy group died within a year after UCBT, with aGVHD being the primary cause of death post-UCBT (**Figure 3B**). Two patients (6.7%) in the No PTCy group and one patient (6.7%) in the 100mg RI-PTCy group relapsed. Of note, two patients in the No PTCy group relapsed after one year and died 1.19 and 1.45 years post-transplant.

## Discussion

Severe aGVHD remains a challenge for successful UCBT (10). With the evolution of transplantation techniques and the application of novel agents over the decades, especially the utilization of PTCy as a GVHD prophylaxis, the optimal GVHD prophylaxis regimen in the UCBT setting may need to be re-determined. To our knowledge, this is the first exploratory study to investigate the preliminary safety and efficacy of RI-PTCy-based GVHD prophylaxis compared to the currently standard CNI-based prophylaxis in adult patients with hematological disorders who received single-unit UCBT.

Delayed engraftment and graft failure are primary concerns of the administration of PTCy in UCBT. In the current study, the median time to neutrophil engraftment was 18 days in the 400 mg group and 16 days in the 100 mg group; for platelet engraftment, it was 31 days and 32 days, respectively, showing no significant difference compared to the No PTCy group. Moreover, no primary or secondary graft failure was observed in the present study. Several studies have reported that the median times for neutrophil and platelet engraftment range from 14 to 23.5 days and 32 to 55 days, respectively (3–6, 20–22). These differences may be attributed to patient characteristics, varying institutional standards of supportive care, and a relatively stronger conditioning regimen. Meanwhile, it was demonstrated that the RI-PTCy may didn’t hamper the engraftment, also indicating that the other transplantation procedures at our institution were well-established. To be noted, the neutrophil engraftment in the 400 mg RI-PTCy group was slightly longer than the other two groups, although without statistical significance. This may be due to fewer ABO identical patients, more bloodstream infections, and a limited patient number. But it still provides us a hint that the 400 mg RI-PTCy may delay the engraftment of neutrophils, suggesting a further randomized controlled trial with a large number of patients is warranted to determine the optimal doses of PTCy.

Transplantation-related mortality after UCBT is the major cause of early death in the previous study (3–6), and high GVHD-related mortality is also observed in our research. The cumulative incidence of grade II-IV aGVHD at day +100 in the No PTCy group was 30.0%, aligning with previous studies that report a range of approximately 20-50% (3–6). Notably, the studies with a higher incidence of aGVHD were conducted several years ago, which also demonstrated the development of transplantation techniques. Impressively, only one patient in the RI-PTCy group experienced grade II-IV aGVHD, underscoring the potential potency of RI-PTCy in preventing aGVHD. However, RI-PTCy has shown no impact on the incidence of PES and ES. It can be explained by the fact that PTCy preferentially reduces the allo-reactive T cells and impairs their functions (23). And the PES was thought to be associated with monocyte proliferation and the release of pro-inflammatory cytokines (24). This phenomenon also supports our data that the engraftment was not hampered by RI-PTCy. Impressively, the 1-year cGVHD cumulative incidence was higher in the 100 mg RI-PTCy group, possibly due to a more intensive conditioning regimen, which is considered a risk factor for cGVHD (25). In short, understanding the dynamics of immune subsets at the very early stage, before and after PTCy use, is crucial for elucidating the mechanism of action of RI-PTCy in UCBT and could offer key insights for clinical practice.

Besides the successful engraftment and preliminary effectiveness in controlling aGVHD with RI-PTCy, early bloodstream infections, CMV/EBV reactivation, VOD, TMA, and HC were also observed. Studies have shown that PTCy in the haplo-HSCT could increase early infections (26), and in our study, the 28-day bloodstream infection rate was highest in the 400 mg RI-PTCy group, compared to the 100 mg RI-PTCy group. Along with a slightly delayed neutrophil engraftment in the 400 mg RI-PTCy group, this suggests that patients may benefit more from PTCy doses lower than 400 mg. But the optimal dosage remains to be determined. The increased endothelial stress observed early after allo-HSCT was primarily due to CNIs and neutrophil engraftment (27, 28). This may explain why the rates of VOD and TMA were similar among the three groups in our study. Furthermore, a relatively higher rate of grade II-IV HC in the RI-PTCy groups was observed, which is in line with other studies (26). Importantly, although long-term outcomes data are still evolving, the RI-PTCy group showed better survival outcomes within a year after UCBT than the No PTCy group, mainly owing to reduced aGVHD-related mortality. Additionally, two patients suffered relapse-related mortality in the No PTCy group, which may be mainly owing to the TP53 mutation (29).

Our study provides valuable preliminary insights showing that RI-PTCy-based GVHD prophylaxis may be more effective at controlling aGVHD, especially in severe cases, than standard CNI-based prophylaxis, while maintaining similar engraftment times and not increasing early infections at 100 mg doses or endothelial dysfunction. However, several limitations were present in the study. First, the sample size was small, and the design was uncontrolled. Therefore, the interpretation of the results requires careful consideration. Surveillance of immune reconstitution and comprehensive long-term outcomes, including the 2-year cumulative incidence of chronic GVHD, GRFS, and NRM, was inadequate and warrants further investigation.

## Conclusion

This pilot study provides initial evidence that RI-PTCy may be a feasible and potentially effective strategy for reducing aGVHD after adult UCBT. The 100 mg dose appeared to offer a more favorable safety profile than the 400 mg dose. However, these findings are preliminary and require confirmation in larger, prospective, randomized controlled trials with sufficient statistical power and longer follow-up. Moreover, the optimal doses and timing must be identified. Additionally, the results emphasize the potential to compare RI-PTCy-based UCBT with PTCy-based haplo HSCT to resolve the debate in selecting alternative donors in the future.

## Data Availability

The original contributions presented in the study are included in the article/supplementary material. Further inquiries can be directed to the corresponding author.
